# Iron Deficiency as a Factor of Worse Prognosis in Patients with Acute Myocardial Infarction

**DOI:** 10.3390/biomedicines13040769

**Published:** 2025-03-21

**Authors:** Aleksander Misiewicz, Krzysztof Badura, Oliwia Matuszewska-Brycht, Jan Krekora, Jarosław Drożdż

**Affiliations:** 2nd Department of Cardiology, Medical University of Lodz, 92-213 Lodz, Poland

**Keywords:** iron deficiency, myocardial infarction, acute coronary syndrome, transferrin saturation, ferritin, cardiovascular disease, reactive oxygen species, oxidative stress

## Abstract

Acute coronary syndromes (ACS) are a leading cause of death and impairment in the adult population. Precise identification and modification of risk factors is crucial for a favorable clinical outcome. In this review, we aim to provide a comprehensive overview of the significance of iron deficiency (ID) in patients with ACS, particularly myocardial infarction (MI). The paper evaluates the impact of ID on the prognosis of ACS patients, highlighting its potential influence on myocardial healing, regeneration and cardiovascular events during the follow-up period. The findings suggest that iron deficiency may have a negative impact on the prognosis of patients with MI, resulting in worse quality of life, physical capacity and higher rehospitalization rates in comparison to patients with normal iron status. Iron supplementation in patients with MI could be beneficial and may have an effect on myocardial healing and left ventricular remodeling.

## 1. Introduction

Cardiovascular diseases are consistently the leading cause of death around the globe. In 2019, they were responsible for almost 18 million of deaths, accounting for 32% of deaths worldwide. The most common diagnoses among this group are myocardial infarction (MI) and stroke [1]. Despite the far-reaching expenditures on treatment and prevention programs, acute coronary syndromes (ACS) still constitute a great burden on healthcare systems and patients themselves. The most common risk factors for coronary artery disease are obesity, hypertension, diabetes, dyslipidemia and nicotinism [2]. In contrast to acute heart failure, the assessment of iron parameters in patients with ACS is not a part of a regular practice, yet iron plays a key role in the transport and storage of oxygen, as well as intracellular respiration. Therefore, its proper concentration and availability seem to be crucial in ischemic processes such as MI. Iron deficiency is the most common deficiency in the human population [3] and its non-invasive diagnosis is based on laboratory markers—ferritin (Fer) and transferrin saturation (TSAT). Values of Fer < 100 µg/L correspond to resource depletion and absolute iron deficiency (ID), and the range of Fer 100–300 µg/L along with TSAT < 20% correspond to functional ID [4,5]. The criteria presented above are widely used in patients with heart failure (HF), with their effectiveness proven in clinical trials [6,7,8]. The aim of this paper is to highlight the importance of ID, its incidence and prognostic value in patients with ACS, as well as to indicate potential areas of development on the basis of the available literature.

## 2. Materials and Methods

**Figure 1:**
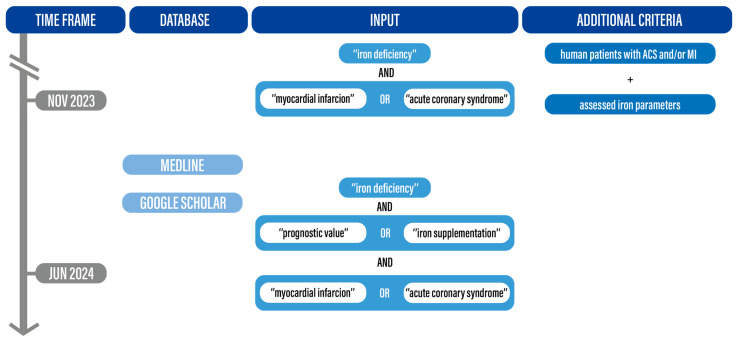
Data collection strategy.

Most scientific papers published to date seem to confirm the negative impact of iron deficiency on the prognosis of patients with myocardial infarction. We evaluated publications on iron deficiency in myocardial infarction written in English. We analyzed MEDLINE and Google Scholar databases between November 2023 and June 2024 using key words and terms, such as: “Iron deficiency” AND (“myocardial infarction” OR “acute coronary syndrome”) AND “iron deficiency” AND “prognostic value” OR “iron supplementation” AND (“myocardial infarction” OR “acute coronary syndrome”). We included studies with human patients with ACS stratified by iron status, in most cases with regard to TSAT and Fer concentration values, with and without anemia, regardless of iron supplementation. We excluded papers which did not answer the question of the review, for example, those which examined only patients with anemia or focused on patients with heart failure. Nine studies, one of which was a systematic review, fulfilled the above-mentioned criteria. The search process is summarized in Figure 1. At the time of this research there were no clinical trials available which assessed the effect of iron supplementation in patients after MI with confirmed ID.

## 3. Pathophysiology of Iron Deficiency in Patients with Coronary Artery Disease

### 3.1. Physiology of Iron Metabolism

In the human body, iron is mostly absorbed within the small intestine through enterocytes. Without parenteral iron supplementation, absorbed iron originates from ingestion of iron-rich foods and oral iron supplementation. Ingested iron is present in the following biological forms: (1) heme-bound Fe^2+^; (2) non-heme Fe^3+^; and (3) Fe^2+^ from oral iron supplementation [9]. Iron solubility increases in the acid mucus within the stomach, where insoluble Fe^3+^, a form normally present in neutral pH, is reduced to soluble Fe^2+^ [10]. Additionally, Fe^3+^ is reduced to Fe^2+^ by ferroreductases localized within the luminal membrane of enterocytes [9]. According to iron solubility, only the reduced form (Fe^2+^) can be transported through biological membranes.

Iron absorption occurs via several pathways. Heme-bound Fe^2+^ is transported to enterocyte cytosol via a heme transporter, whereas Fe^2+^ is transported through the divalent metal transporter 1 (DMT1). Within the enterocyte, oxidation occurs, which is catalyzed by heme-oxidase and leads to porphobilinogen formation and Fe^2+^ release. In cytosol, Fe^2+^ may undergo oxidation to Fe^3+^ again with subsequent binding with Fer, which constitutes a main iron storage molecule and its localized mostly in cytosol; however, mitochondrial and nuclear forms have also been reported [9,10].

To fulfill metabolic needs, Fe^2+^ can be transported into blood via ferroportin 1 (FPN1). Within the bloodstream, iron is transported by transferrin, which constitutes a specialized protein with an ability to bind the Fe^3+^ form. Thus, Fe^2+^ is converted to Fe^3+^ by hephaestin [9,10], before binding with transferrin. Iron release from enterocytes is regulated by hepcidin, an acute-phase protein secreted by the liver in response to inflammation. Hepcidin leads to FPN1 endocytosis with subsequent ubiquitination and FPN1 lysis.

Apart from the absorption and iron release from enterocytes, iron homeostasis is maintained by peripheral iron intake. Fe^3+^-saturated transferrin (Tf) binds with transferrin receptor 1 (TfR1), which is followed by endocytosis. Fe^3+^ is released and reduced within endosomes to Fe^2+^ which can be used in biological processes, e.g., enzyme synthesis or oxygenated again and stored by Fer.

### 3.2. Etiology and Pathogenesis of Iron Deficiency in Cardiovascular Disease

ID occurs in approximately 50% of patients with established cardiovascular disease. Moreover, up to 60% of patients with coronary artery disease have been shown to suffer from ID [11]. Recent data indicate that systemic iron deficiency, even without anemia, worsens mortality and prognosis in acute and chronic heart diseases [12]. Several factors contribute to ID in CVD, comprising enhanced inflammation, malnutrition and comorbidities such as heart failure leading to kidney dysfunction and reduced iron absorption within the intestine [11,13]. The consequences of iron deficiency at the cellular level include mitochondrial dysfunction, the impairment of non-mitochondrial energy production pathways, a reduction in myoglobin and oxygen stores, an increase in reactive oxygen species (ROS), an increase in abnormalities during replication and repair of the cell DNA, and an increase in abnormal immune responses [14], which further contribute to tissue damage caused by ischemia [15,16,17].

### 3.3. Iron Deficiency and Ischemia-Related Cardiomyocyte Damage

Iron content within cardiomyocytes is highly dependent on influx and outflow balance, which is mainly maintained by TfR1, DMT1 and the hepcidin–ferroportin axis [18]. Iron importation is enhanced due to the activation of TfR1 and DMT1 in iron-deficient cardiomyocytes, whereas the transcription of ferroportin (FPN) is decreased. Moreover, hepcidin, whose expression is increased in ID, is responsible for FPN inhibition and degradation. Finally, intracellular iron storage is decreased with the aim to increase the cytosolic iron pool via the decreased expression and increased degradation of ferritin [12].

The potentially poor outcomes of patients with ACS and concomitant ID (see Section 3.1) may be related to metabolic changes that occur in myocardial ID. ID may be associated with enhanced oxidative stress, as it constitutes an essential component of ROS-neutralizing enzymes. Endothelial nitric oxide synthase (eNOS) and soluble guanylate cyclase (sGC) belong to the eNOS/sGC/protein kinase-G (PKG) pathway and contain protoporphyrin IX, which includes iron within its structure [12]. The decreased synthesis of eNOS and sGC and subsequent eNOS/sGC/PKG pathway inhibition may be associated with enhanced oxidative stress after reperfusion, due to ischemia–reperfusion injury [19,20]. This phenomenon may explain the larger infarct size and decreased contractility of iron-deficient cardiomyocytes.

ROS constitute well-known factors leading to cellular damage. This phenomenon also involves cardiomyocytes, where cardiac troponin release is associated with redox state dysregulation due to enhanced ROS formation and decreased degradation. Oxidative stress leads to cardiomyocyte maladaptation due to electrophysiological and mechanical impairment.

Electrophysiological impairment is associated with ROS-mediated function reverse of the Na^+^/Ca^2+^ exchanger (NCX), and increased influx of Ca^2+^ through Ca^2+^ L-type channels. Moreover, oxidative stress affects the sarcoplasmic reticulum (SER), which is involved in calcium balance maintenance. Oxidative stress decreases sarcoplasmic reticulum Ca^2+^-adenosine triphosphatase 2 (SERCA2) function, leading to decreased Ca^2+^ influx to SER, whereas the function of ryanodine receptor 2 (RyR2) is enhanced [21]. These processes lead to calcium overload. It should be noted that an increased Ca^2+^ level within cardiomyocytes, often associated with impaired function of NCX and RyR2, may lead to malignant arrythmias via a mechanism of triggered activity: early depolarizations and delayed afterdepolarizations [22].

Mechanical function impairment of cardiomyocytes, manifesting as impaired systolic and diastolic function, is mainly caused by an enhanced formation of the tissue inhibitor of metalloproteinases and a decrease in metalloproteinase synthesis, which lead to myocardial fibrosis. Moreover, calcium overload is associated with decreased sensitivity of myofilaments which leads to impaired contractility [21].

**Figure 2:**
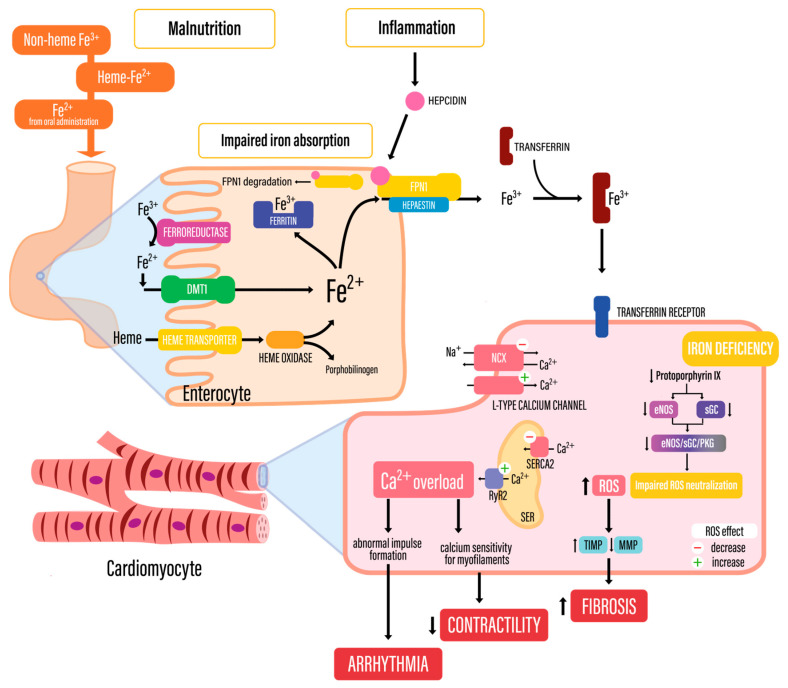
Iron metabolism and mechanisms of cardiac damage in iron deficiency. DMT1—divalent metal transporter 1; FPN1—ferroportin 1; Na^+^/Ca^2+^ exchanger; MMP—metalloproteinase; ROS—reactive oxygen species; RyR2—ryanodine receptor 2; SER—sarcoplasmic reticulum; SERCA2—sarcoplasmic reticulum Ca^2+^-adenosine triphosphatase 2; TIMP—tissue inhibitor of metalloproteinase.

Figure 2 summarizes the pathophysiological mechanisms leading to cardiac dysfunction in enhanced oxidative stress.

## 4. Diagnosis

It has been shown that preexisting anemia occurs in 27.7% of patients presenting with ACS and constitutes a risk factor of all-cause mortality [RR 2.08 (95% CI 1.70–2.55)] [23]. Recent World Health Organization guidelines define cutoffs to diagnose anemia in individuals between 15 and 65 years old as <12 g/dL for non-pregnant women and <13 g/dL for men. The simplified definition commonly used in clinical practice defines anemia as <12 g/dL in women and <13 g/dL in men.

It should be noted that iron deficiency may be present with or without concomitant anemia. In isolated ID, Hb levels persist within the normal range.

ID can be diagnosed following two diagnostic pathways: invasive and non-invasive. As the invasive pathway is based on bone marrow biopsy, it is rarely used in clinical practice due to a higher risk of complications when compared to standardized laboratory tests using blood serum. Thus, the non-invasive pathway is preferred in clinical practice, whereas a bone marrow biopsy should be performed only after unclear laboratory test results for a definitive diagnosis [24]. To date, two laboratory parameters are supported by clinical studies to reflect iron status in cardiovascular diseases—Fer and TSAT. Serum Fer, secreted by macrophages, Kupfer cells and hepatocytes have an ability to carry labile extracellular iron. Levels of serum Fer are decreased in absolute iron deficiency. TSAT represents the calculated total serum iron/total iron binding capacity (TIBC) × 100. TIBC can be calculated as transferrin × 1389.

**Figure 3:**
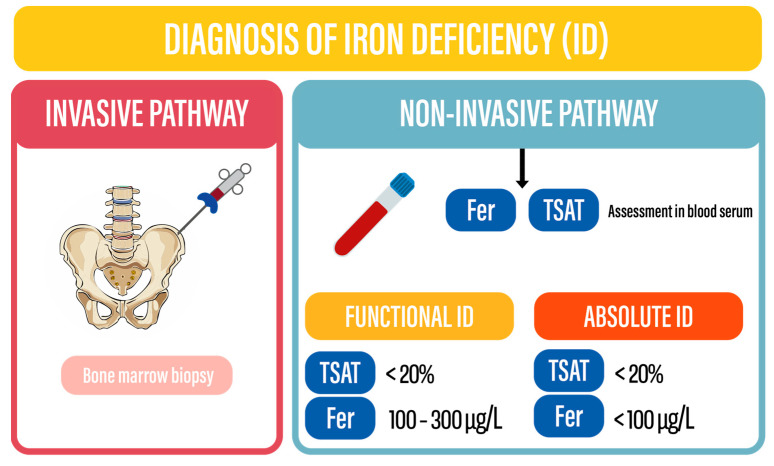
Diagnosis of iron deficiency. Fer—ferritin; ID—iron deficiency; TSAT—transferrin saturation.

In order to assess the iron status of patients with CVD, both Fer and TSAT from blood serum should be assessed. Fer < 100 mcg/L allows clinicians to diagnose absolute iron deficiency, whereas Fer levels between 100 and 300 mcg/L associated with decreased TSAT < 20% suggest functional iron deficiency. It should be noted that Fer concentrations and TSAT may be influenced by several factors. Moreover, different cutoffs of each parameter may be applicable according to comorbidities such as chronic kidney disease or liver cirrhosis [25]. The iron status assessment pathways have been presented in Figure 3.

## 5. Iron Deficiency and Prognosis in Acute Coronary Syndromes

The data included in the present review resulted from observational, mainly prospective studies. The main characteristics of and differences between cited original studies can be found in Table 1.

The study by Cosentino et al. [26] assessed the iron status of consecutive patients with ST-elevated myocardial infarction (STEMI) undergoing percutaneous coronary angioplasty. The iron-deficient group of patients accounted for 56% of the population and was characterized by a higher prevalence of type 2 diabetes, anemia, higher concentrations of high-sensitivity cardiac troponins and circulating mitochondrial DNA on admission. Cardiac magnetic resonance imaging (CMR) did not reveal significant differences between patients with and without ID pertaining to left and right ventricular volumes and ejection fractions. Patients with ID had a larger area at risk, a similar infarct size, and, as a result, a higher myocardial salvage index in comparison to patients with a normal iron status. Paradoxically, patients with ID had a better in-hospital course and a lower incidence of the primary composite endpoint consisting of in-hospital mortality and Killip–Kimball class ≥ 3, even after adjustment for major confounders [odds ratio (OR) 0.50, 95% confidence interval CI 0.27–0.93; *p* = 0.02] [26]. Analogical results had been drawn in a post hoc substudy based on the CULPRIT-SHOCK trial—a randomized multicenter study, which included patients with AMI treated with percutaneous intervention (PCI), complicated by cardiogenic shock. In this case, ID without anemia was associated with a lower probability of death from any cause or renal replacement therapy [OR 0.63 (CI 0.31–1.08); *p* = 0.06] during a 30-day follow-up period in comparison to nonanemic patients with a regular iron status [27].

Contrary to the above-mentioned findings are the results from the research conducted by Fujinaga et al. [28], in which 352 nonanemic patients with STEMI who underwent successful PCI were stratified by serum iron concentration on admission. Patients with low iron concentration (<70 mg/dL) constituted 48% of the population and were older, more frequently women (36% vs. 17%, *p* < 0.001), had elevated C-reactive protein (CRP) (31 vs. 12%, *p* < 0.001), higher Killip–Kimball class (>II; 30 vs. 20%, *p* = 0.04), higher plasma BNP (286 ± 315 vs. 184 ± 227 pg/mL, *p* = 0.001) and higher incidence of in-hospital death (6.5 vs. 1.6%, *p* = 0.03) in comparison to those without ID. LVEF was similar in both groups [28].

Another study with a short-term observation period was conducted by Merono et al. [29] and aimed to test patient-assessed quality of life (QOL) and exercise capacity among patients with ACS. Iron deficiency was found in 57% of patients included in the study at baseline and persisted in 46% of the population on the 30th day of follow-up. The patients with ID were older, had lower hemoglobin concentrations and higher inflammatory parameters. Exercise tolerance in the group with ID was lower in both the Bruce protocol treadmill test and the 6 min walk test compared to the patients without ID (7.9 ± 2.9 vs. 9.3 ± 2.6 METS; *p* = 0.003; 277 vs. 423 m; *p* = 0.009, respectively). Similarly, patients’ perceived QOL in the ID group was much lower (OR: 1.9; 95% CI: 1.1–3.3; *p* < 0.001). Prior aspirin intake was independently associated with ID [OR 3.254 (CI 1.373–7.716); *p* = 0.007]. The results of the experiment showed no correlation between iron deficiency and increased cardiovascular morbidity or mortality during short-term follow-up [29].

However, these outcomes do not indicate that ID does not affect the long-term prognosis of ACS patients. In the study by Silva et al. [30], consecutive patients with ACS were included. The ID group accounted for 36% of the study population and consisted of older patients with a higher prevalence of comorbidities, and in whom coronary artery bypass grafting was more common, in comparison to patients without ID. Patients with ID had worse baseline clinical conditions, characterized by an increased prevalence of higher Killip–Kimball class (class II-IV 21.8% vs. 12.1%, respectively, *p* < 0.001), anemia (20.1% vs. 8.5%, respectively, *p* < 0.001), glomerular filtration rate below 60 mL/min (30.2% vs. 17.9%, respectively, *p* < 0.001), triple-vessel disease (26.6% vs. 16.5%, respectively, *p* < 0.001), and left ventricular ejection fraction below 40% (34.2% vs. 22.3%, respectively, *p* < 0.001). Follow-up time averaged 738.77 days, during which patients were evaluated for all-cause mortality, death from cardiovascular causes, occurrence of stroke, reinfarction, heart failure exacerbation (New York Heart Association [NYHA] class III and IV), angina (Canadian Cardiovascular Society class > I) and rehospitalization. The group with ID had a higher incidence of the aforementioned adverse events, notably higher any-cause mortality (12.6% vs. 6.3%, *p* = 0.004), more frequent heart failure exacerbation (10.5% to 5.3%, *p* = 0.011) and rehospitalization (13.7% vs. 9.8%, *p* = 0.048) compared to controls. Moreover, the patients burdened with ID had a higher all-cause mortality rate regardless of the presence of anemia [30].

Another publication examining the prognosis of patients with ID was the study by Gonzales-D’Gregorio et al. [31], which focused on the follow-up of patients aged > 65 years with ACS (STEMI, NSTEMI or UA). TSAT and ferritin were stratified into quartiles yielding the following ranges for TSAT: Q1TSAT: 2.8–11.8% (n = 63), Q2TSAT: 11.8–17.2% (n = 63), Q3TSAT: 17.2–24.6% (n = 63), Q4TSAT: 25–95% (n = 63); for ferritin: Q1fer: 9–81 ng/mL (n = 63), Q2fer: 82–147 ng/mL (n = 63), Q3fer: 148–258 ng/mL (n = 63), Q4fer: 259–1609 ng/mL (n = 63). The median follow-up was 4.7 years, during which 121 (48%) patients died. Patients in the lower quartiles of TSAT were more likely to suffer from STEMI and had higher NT-proBNP serum concentrations on admission. There were no significant differences in frailty index (Fried score), GRACE score, Charlson Comorbidity Index, left ventricular systolic function or management strategy between the patients across the ferritin and TSAT quartiles. After multivariate adjustment, TSAT as a continuous variable was inversely and nonlinearly associated with the risk of long-term mortality (*p* = 0.002), with an exponential increase in risk from values of 20% and below. Lower TSAT values (Q1TSAT vs. Q2–Q4TSAT) were found to be strongly associated with the risk of death in anemic patients (HR: 2.13; 95% CI: 1.31–3.48; *p* = 0.002), but had a neutral effect in nonanemic subjects (HR: 0.80; 95% CI: 0.36–1.70; *p* = 0.539). Ferritin serum concentrations and the current definition of ID were not independently associated with mortality in this paper [31].

In the study conducted by Zeller et al. [5], iron deficiency was present in 29% of 836 participants with ACS, more common in women (42.8%, *p* < 0.001) and co-present with anemia (42.5%, *p* < 0.001). Both studied iron parameters positively correlated with male gender, dyslipidemia and troponin I serum concentration. During a mean follow-up time of 4 years, 111 patients died of cardiovascular reasons or had non-fatal myocardial infarction. ID was an independent predictor for both endpoints after adjustment for age, sex and cardiovascular death risk factors (HR 1.52; 95% CI: 1.03–2.26; *p* = 0.037) [5].

Similar conclusions were drawn by Jenča et al. [32] in a prospective observational cohort study. A total of 1156 consecutive patients with no previous history of coronary artery disease and diagnosed type I MI were included. Blood samples were collected in the morning on the first day after hospital admission and serum iron concentration, ferritin, transferrin, soluble transferrin receptor (sTFR), TIBC and TSAT were determined. The median follow-up time amounted to 1224 days (IQR 626–1782), during which 194 (16.8%) patients died. Each evaluated ID criterion, except for ferritin, was independently associated with all-cause mortality.

Due to conflicting results from formerly published papers on the issue of ID in ACS, researchers have suggested new criteria, named “Prague ID”, based on serum iron concentration and sTfR. The combination of iron ≤ 12.8 µmol/L and sTfR ≥ 3.0 mg/L showed the best association with total mortality risk, also after adjustment for the GRACE score and other confounders [32].

There is only one systematic review on the prognostic value of ID in patients with ACS available to date. Reinhold et al. [33] summarized seven papers, three of which were conference abstracts. According to their findings, ID was present in 43% (n = 1226) of all patients with ACS and was associated with worse patient prognosis during mid- to long-term follow-up. Due to the differences in outcome measures and follow-up periods of each analyzed study, a meta-analysis could not be performed. Conclusions drawn from this research are similar to ones already mentioned in this paper, due to being based on similar source data [33].

**Figure 4:**
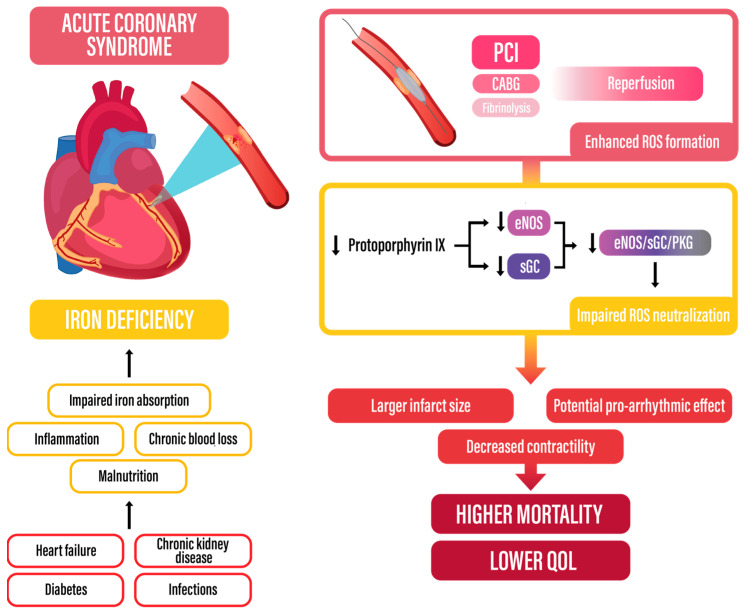
Iron deficiency in acute coronary syndromes—summarizing figure. CABG—coronary artery bypass grafting; eNOS—endothelial nitric oxide synthase; PCI—percutaneous coronary intervention; PKG—protein kinase G; ROS—reactive oxygen species; sGC—soluble guanylate cyclase.

Figure 4 summarizes factors leading to iron deficiency in ACS patients and potential mechanisms that may influence prognosis.

## 6. Iron Supplementation and ACS

In light of the available scientific literature, no clinical trials have been established to date which investigate the effect of iron supplementation in ACS patients with known ID. There was an experiment conducted by Florian et al. [16] to test the effect of iron supplementation in post-MI patients, known as the NIMINI-3 trial. The prospective non-randomized unblinded study involved 39 patients with established STEMI undergoing primary percutaneous coronary intervention with successful reperfusion, of which 17 were assigned to the group receiving parenteral iron in the form of ultrasmall superparamagnetic iron-oxide (USPIO) and 22 to the group receiving placebo. Patients underwent myocardial MRI up to 1 week and 3 months after ACS. Iron administration occurred within 24 h after the imaging was performed. During follow-up, none of the patients had relevant cardiovascular events. A significant increase in left ventricular ejection fraction was observed in both groups, with a greater effect in the iron-treated group (USPIO: 53 ± 10%–59 ± 9%; *p* = 0.002; control: 54 ± 6%–57 ± 10%; *p* = 0.005). In both groups the infarct size was significantly reduced in the follow-up MRI with a predominance of the study group (USPIO: 27 ± 8% to 17 ± 9%; *p* < 0.001 vs. control: 27 ± 12% to 20 ± 11%; *p* < 0.001). It seems that USPIO-based intravenous iron administration in patients with STEMI is associated with more favorable healing and left ventricular remodeling. The limitations of the study were the small patients groups and the lack of iron parameter analysis and ID diagnosis [16]. The results of this experiment seem to be supported by the findings of Mohanty et al. [34] and Huang et al. [17] which have shown a positive, statistically significant correlation between serum iron concentration and LVEF improvement 6 months after STEMI/NSTEMI [17,34]. Deeper relationships between cardiac remodeling and ischemia are the subject of research in the work of Inserte et al. [35] The paper reports on a prospective observational study on humans and an interventional experiment on mice. In total, 141 patients, who were tested for their iron status with their first anterior STEMI, were treated with PCI and underwent CMR at the beginning and at the end of the 6-month observation period. ID was defined according to the current consensus. Patients with ID had significantly larger mean infarct size and more extensive microvascular obstruction than those with normal iron status (*p* < 0.05). They also showed a greater increase in LV volume at the end of the follow-up, with similar changes in LVEF. Adverse LV remodeling occurred in 37.8% of patients with ID and 14.1% of those without ID (*p* = 0.004). The pathophysiological background was tested in the second part of the study, where mice were parted into two groups, based on a 4-week diet period. Some mice in the study group were given intravenous iron supplementation. Mice were subjected to MI by left descending coronary artery ligation. The myocardium of mice fed with an ID diet was subdued to increased oxidative and nitrosative stress, significantly reducing the antioxidant capacity in comparison to controls (*p* = 0.008). The infarct size was significantly larger in the study group (58.1% ± 3.0% vs. 40.4% ± 3.7% of area at risk; *p* = 0.004). Iron supplementation prevented the above-mentioned issues [35].

## 7. Discussion

ID is a common condition in patients with acute coronary syndrome. The results of the papers summarized in our study seem to be contradictory. The influence of ID on short-term prognosis according to the current data is ambiguous. Research conducted by Cosentino et al. [26] and Obradowic et al. [27] included highly specific groups of patients—with STEMI and with ACS and multivessel disease complicated by cardiogenic shock, respectively. Iron parameters were assessed either by admission or during coronarography. Important to note is the fact that TSAT and Fer are both reactants whose concentrations vary during acute conditions. Transferrin saturation decreases, but ferritin, as an intracellular protein, increases its presence in circulation after cellular damage [36]. In this case, low ferritin concentration may relate to less myocardial damage, and consequently, better short-term prognosis, which would also be supported by Jenca et al. [32] Taking into consideration the limited group of patients and early iron parameter assessment in these two papers, conclusions drawn pertaining to better prognoses during short-term follow-up should be regarded with caution. The findings of Fujinaga et al. [28] are available only in abstract form, in which data contributing to the study population, recruitment and methods are strictly limited, and the sole examination of iron serum concentration decreases the impact of the presented results. Only in the study by Merono et al. [29] were iron parameters assessed 5 days after ACS, during initial stabilization. Consecutive patients were recruited, but only those who had undergone the above-mentioned iron examination were further evaluated. ID had no effect on mortality in short-term follow-up, but was associated with worse QOL and exercise tolerance, which may relate to the increased prevalence of chronic diseases and, due to this, possible recurrent inflammation in this group, which impedes iron absorption and affects regular serum iron concentrations [37,38]. In consequence, it may lead to increased ROS concentration and impede heart muscle contractility, due to calcium influx and its overload in cardiomyocytes.

However, current evidence supports the importance of ID as a factor of worse prognosis in patients with ACS. Papers assessing mid- to long-term follow-up seem to agree on ID in relation to the increased occurrence of major adverse cardiovascular events regardless of age, gender and other comorbidities. In research conducted by Silva et al. [30] and D’Gregorio et al. [31], lower TSAT was associated with age and an increased incidence of comorbidities. ID was more common in patients suffering from STEMI and who had higher concentrations of hsCRP, which also supports the thesis of iron being an anti-inflammatory agent reducing atherosclerosis [39]. Iron acts as a protective agent in cardiomyocytes—increasing overall antioxidative capacity, reducing adverse effects of hypoxia and diminishing cellular damage [40]. Increased calcium concentration in cardiomyocytes during ID conditions is related to increased fibrosis, which in the long term could lead to adverse LV remodeling. In the work by Jenca et al. [32], newly developed PragueID criteria based on soluble transferrin receptor and iron serum concentrations showed the best association with total mortality risk in comparison to TSAT and Fer. Their presence in blood serum appeared to be more stable during acute conditions, which could facilitate more reliable ID diagnosis. This new criteria may also be used in the healthy population, as ID in this group has been proven to be a major risk factor associated with increased all-cause mortality [41].

At this moment in time, there are not enough data to support regular iron supplementation in patients with ACS with confirmed ID, even though we already have some evidence that it may be related to better tissue healing and LV remodeling prevention [16]. In the FAIR-HF trial, more than 60% of the patients included suffered from heart failure caused by ischemic heart disease. The administration of intravenous ferric carboxymaltose was beneficial in all cases regardless of the hemoglobin concentration [7]. The potentially positive effect of iron supplementation could also be supported by necessary pharmacotherapy after ACS, namely dual antiplatelet therapy, which is a major risk factor for potential bleeding and cause of—naturally—iron deficiency [42].

## 8. Conclusions

ID is a common condition in patients with ACS. Although its influence on short-term follow-up appears to be uncertain, ID seems to be independently associated with worse patient prognosis during mid- to long-term follow-up. Transferrin saturation, contrary to ferritin, is not upregulated during acute conditions, which may be crucial in the non-invasive diagnosis of ID. TSAT appears to have a greater predictive value of death and other adverse events compared to ferritin or serum iron concentration, but there are new, promising, precise markers used for non-invasive ID diagnosis in the works. Iron supplementation in ACS patients may have a beneficial effect on myocardial healing and left ventricular remodeling after myocardial infarction, but further research, especially on human models, is needed to support this relationship.

## Figures and Tables

**Table 1:** A review of original publications examining iron deficiency in a population with ACS—short-term follow-up.

Author	Cosentino N [26]	Obradovic D [27]	Fujinaga H. (Abstract Only) [28]	Merono O [29]	Silva C [30]	González-D’Gregorio J [31]	Zeller T [5]	Jenča D [32]
Year	2019	2024	2013	2016	2021	2018	2018	2024
Study type	prospective observational	randomized trial	prospective observational	prospective observational	retrospective	prospective observational cohort	prospective observational cohort	prospective observational cohort
Population	420	427	352	244–>226	817	252	836	1156
ID (%)	237 (56%)	72 (~17%) [patients with ID without anemia]	169 (48%)	139–>102 (57%–>46%)	298 (36%)	n/s	243 (29.1%)	357 (31%)
Observation time	ACS-related hospitalization	30 days	ACS-related hospitalization	30 days	2 years	IQR: 2–5.4 years	4 years	1224 days
Conclusions	ID in ACS is associated with better in-hospital prognosis [OR 0.50 (CI 0.27–0.93)].	ID in AMI complicated by cardiogenic shock is associated with lower probability of death from any cause or renal replacement therapy [OR 0.63 (CI 0.31–1.08)].	ID on admission is associated with elevated CRP and higher Killip–Kimball class, and predicts poor outcomes after primary PCI in nonanemic STEMI patients.	ID is associated with worse exercise tolerance [OR 2.9 (CI 1.4–5.5)] and lower quality of life [OR: 1.9 (CI: 1.1–3.3)] but has no effect on mortality during short-term follow-up.	ID is an independent predictor of death / development of heart failure in ACS patients [HR 1.66 (CI 1.11–2.50)].	Lower TSAT levels are independently associated with an increased risk of long-term mortality [HR 1.54 (CI 1.03–2.30)].	Iron deficiency is associated with unfavorable medium-term outcomes (independent of systolic heart function), the extent of myocardial necrosis and anemia [HR 1.52 (CI 1.03–2.26)].	Iron deficiency is a common burden in patients with first ACS without past CAD history and, depending on measured parameters, is associated with all-cause mortality.

ACS—acute coronary syndrome; AMI—acute myocardial infarction; CAD—coronary artery disease; CI—confidence interval; HR—hazard ratio; ID—iron deficiency; IQR—interquartile range; n/s—not specified; OR—odds ratio; PCI—percutaneous intervention; STEMI—ST-elevated myocardial infarction; TSAT—transferrin saturation.

## Abbreviations

The following abbreviations are used in this manuscript:

ACS	acute coronary syndrome
AMI	acute myocardial infarction
CABG	coronary artery bypass grafting
CAD	coronary artery disease
CI	confidence interval
CMR	cardiac magnetic resonance imaging
CRP	C-reactive protein
CVD	cardiovascular diseases
DMT1	divalent metal transporter 1
eNOS	endothelial nitric oxide synthase
EF	ejection fraction
Fer	ferritin
FPN	transcription ferroportin
FPN1	transcription ferroportin 1
HF	heart failure
HR	hazard ratio
ID	iron deficiency
IQR	interquartile range
LV	left ventricle
LVEF	left ventricular ejection fraction
MI	myocardial infarction
MMP	metalloproteinase
NCX	Na^+^/Ca^2+^ exchanger
n/s	not specified
NSTEMI	non-ST-elevated myocardial infraction
NT-proBNP	N-terminal pro B-type natriuretic peptide
NYHA	New York Heart Association
OR	odds ratio
PCI	percutaneous intervention
PKG	protein kinase-G
QOL	quality of life
ROS	reactive oxygen species
RR	risk ratio
RyR2	ryanodine receptor 2
SER	sarcoplasmic reticulum
SERCA 2	sarcoplasmic reticulum Ca^2+^-adenosine triphosphatase
sGC	soluble guanylate cyclase
STEMI	ST-elevated myocardial infarction
sTFR	soluble transferrin receptor
Tf	transferrin
TfR1	transferrin receptor 1
TIBC	total iron binding capacity
TIMP	tissue inhibitor of metalloproteinase
TSAT	transferrin saturation
UA	unstable angina
